# Insights in Neuropsychiatry: Suicide and Self-Mutilation in the Mena Region- a Bibliometric Quantitative and Co-occurrence Medline-Based Analysis

**DOI:** 10.7759/cureus.18680

**Published:** 2021-10-11

**Authors:** Elyas Wakim, Said El Hage, Steven Safi, Antonio El Kareh, Jad El Masri, Pascale Salameh

**Affiliations:** 1 Orthopaedics, Faculty of Medical Sciences - Lebanese University, Hadath, LBN; 2 General Medicine, Faculty of Medical Sciences - Lebanese University, Hadath, LBN; 3 Neurosciences, Faculty of Medical Sciences - Lebanese University, Hadath, LBN; 4 Epidemiology and Public Health, Faculty of Medical Sciences - Lebanese University, Hadath, LBN; 5 Public Health, Institut National de Santé Publique, d'Épidémiologie Clinique et de Toxicologie – Liban, Beirut, LBN; 6 Public Health, University of Nicosia Medical School, Nicosia, CYP; 7 Pharmacy, Faculty of Medical Sciences - Lebanese University, Hadath, LBN

**Keywords:** research activity, mental health, arab world, bibliometric analysis, self-injury, self-mutilation, suicide

## Abstract

Background

Little is known about self-mutilation and suicide-related research in the Arab world.

Aim

We aim to quantify research activity related to suicide and self-mutilation, according to socio-economic factors, and assess inter-regional collaborations and trends of topics in the Arab world in the last 16 years.

Methods

A search was conducted through the PubMed database to find articles related to suicide and self-mutilation, according to author affiliation in the 22 Arab countries between 2004 and 2019 (inclusive), and standardized according to mean population, suicide, and self-mutilation disability associated life years (DALY), and gross domestic product (GDP). VOS Viewer was used for keyword and organization co-occurrence analysis.

Results

Only 0.61% of articles related to suicide and self-mutilation published between 2004 and 2019 were of Arab origin, compared to 1.76% in South America and 7.94% in Far East Asia. Suicide and self-mutilation articles make up 0.09% of the total publications in the Arab region. Egypt, Saudi Arabia, and Lebanon had the highest number of published articles related to suicide and self-mutilation. When comparing publications per million persons, Lebanon and Kuwait ranked first with 5.15 and 3.40 publications per million persons. Lebanon showed the highest number of publications per USD billion GDP, with 0.75 publications. The highest number of publications per 1% self-injury-related DALY was recorded in Saudi Arabia, with 46.97 publications. In recent years, VOS Viewer revealed poor inter-regional collaborations and a modest but increasing trend towards depression, poisoning, and cross-sectional studies.

Conclusion

Despite increasing publications, the region still lags in terms of suicide and self-mutilation research activity. A pan-Arab strategy should be implemented to refine suicide-related research and increase mental health awareness.

## Introduction

It is estimated that 800,000 people commit suicide every year. To put it into perspective, there is one case of suicide every 40 seconds. According to World Health Organization (WHO), suicide ranks the 18th leading cause of death [1].

The total number of suicide deaths has increased by 6.7% from 1990 to 2016 [2], and a recent study even suggested that the actual number of suicide is most probably underestimated [3].

Suicidal behaviour disorder is defined by the Diagnostic and Statistical Manual of Mental Disorders (DSM-5) as an attempted suicide in the last two years, excluding suicidal ideation and non-suicidal self-injurious behaviour. In contrast, suicidal ideation is characterized by thinking about or planning suicide. Self-mutilation, also known as deliberate self-injury and Non-Suicidal Self-Injury (NSSI), is defined as the act of deliberately harming your own body as a way to cope with intense anger and emotional pain [4]. Studies show a correlation between personality traits and suicidal ideation, while others show genetic predisposition along with depression [5]. Methods of suicide range in lethality; thus, people can be categorized into attempters and completers [6]. As the name suggests, suicide completers use lethal techniques, hanging the most common method, whereas suicide attempters commonly use drug poisoning and stabbing; these methods are non-lethal in 70% of cases [6]. Literature shows that for each adult death by suicide, there are, on average, more than 20 attempts [1]. Techniques of suicide also differ according to gender, region, culture, and availability of the suicidal tool.

On the other hand, half of NSSI reported multiple methods such as cutting, biting, burning, scraping skin, hitting, and interfering with wound healing [7]. Although self-harm is not a suicidal attempt, there is no suicidal ideation when performing self-mutilation; they both share the same risk factors [8]. Klonsky et al. uncovered a higher correlation between NSSI and suicidal attempts compared to the already established suicide risk factors, including depression, anxiety, impulsivity, and borderline personality disorder [9].

The Arab world has witnessed conflicts, wars, revolutions, and terrorism. Youth unemployment rates are of the highest worldwide and reached a staggering rate of 20% in the region, noting a severe heterogeneity in the results causing females to suffer from 15.6% unemployment [10]. These results shed light on gender inequality, a challenging socio-economical problem in the Arab world, a region with one the highest gender gap score in the world. Moreover, diverse socio-cultural challenges are often encountered in health-related settings, especially mental health problems [11].

Little is known about the activity related to suicide and self-mutilation in the 22 Arab countries. In the last ten years, research productivity improved by 160% in the Arab world [12]. Keeping these facts in mind, it is in our interest to look into the trends of mental health diseases in the Arab world, notably suicide. Nevertheless, due to the stigmatization of mental problems in general and suicide in particular among monotheist religions, suicide or the attempt to do so is associated with weakness and even shame in most Arab countries [13]. Thus, we hypothesize that research related to suicide is low in the region. This study aims to quantitatively assess the Arab world's research output devoted to suicide and self-mutilation and its worldwide contribution in the scientific literature, normalizing results by several socio-economic factors such as GDP, mean population, and suicide and self-mutilation DALYs. In addition, the authors evaluated collaborations between Arab institutions and topic trends in the study's time frame.

## Materials and methods

This study is a bibliometric analysis assessing suicide and self-mutilation in the Arab world. A bibliometric analysis evaluates the number of publications in a certain area in a quantitative and explorative manner to assess research output and impact in literature.

Database

Access to the Medline database was through PubMed, provided by the National Library of Medicine, the world's largest biomedical database. Medical Subheadings (MeSH) are used article indexing, where each article is identified by specific MeSH terms related to its subject.

First of all, the search for articles discussing suicide and self-mutilation has been conducted in the 22 Arab countries, between the period of 2004 till 2019, and included: Algeria, Bahrain, Comoros, Djibouti, Egypt, Iraq, Jordan, Kuwait, Lebanon, Libya, Mauritania, Morocco, Oman, Palestine, Qatar, Saudi Arabia, Somalia, Sudan, Syria, Tunisia, United Arab Emirates, and Yemen.

Moreover, we calculated the average Growth Domestic Product (GDP) in billion United States Dollars (USD), average population, and DALYs related to self-harm for each of the countries mentioned above [14-16]. Including these factors in the analysis leads to a standardized comparison between countries by dividing the number of articles published for each Arab country by its average GDP, population, and percentage of self-harm-related DALY.

It is worthy to note that according to the International Classification of Diseases version 10 (ICD-10), suicide and intentional self-harm were both included in the self-harm DALY category of the Global Burden of Diseases (GBD).

Search strategy

**Figure 1:**
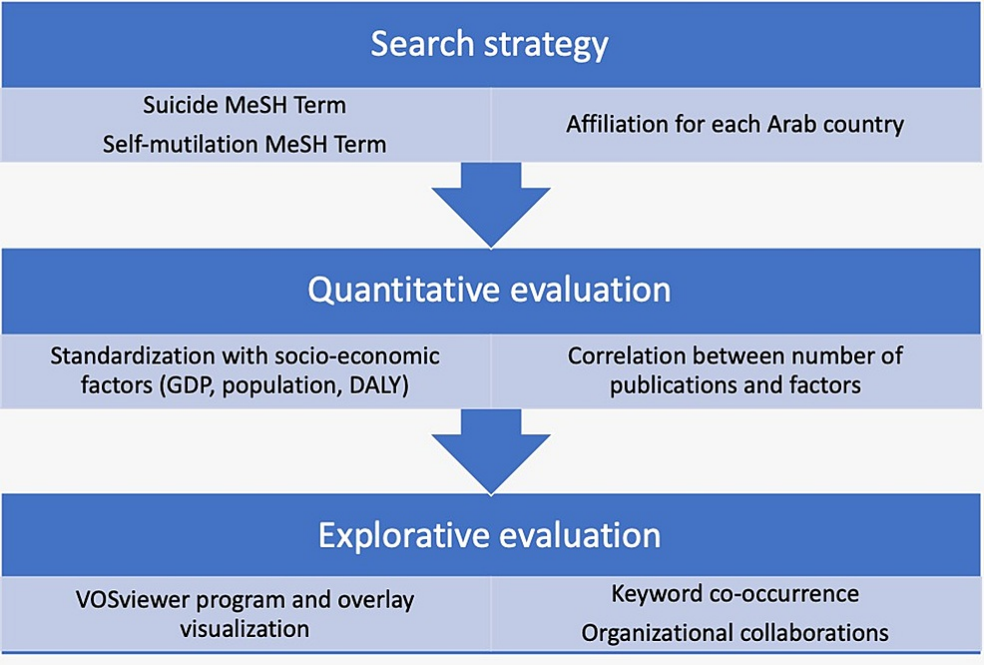
Algorithm-based visual representation of the search strategy and methodological approach for suicide-research assessment in the Arab world MeSH- Medical Subject Headings; DALY- Disability-adjusted life year; GDP- Gross domestic product

It is important to note that MeSH terms are of hierarchal organization. A search for suicide [MeSH term] would retrieve articles about suicidal ideation, attempt, and total related articles. Therefore, we used both "suicide" and "self-mutilation" MeSH terms in our search. Boolean operators (AND, OR, NOT) and affiliation of authors ([affiliation]) were used in our search to indicate the country of publication and the correlation between terms. For example, for articles published in Somalia, we used the following entry: (("suicide"[MeSH Terms]) OR (("self-mutilation"[MeSH Terms]) AND (("2004"[Date - Publication]: "2019"[Date - Publication]))) AND (Somalia [Affiliation]). For further sectionalization, "suicide attempted," "suicide completed," "suicidal ideation," and "suicidal assisted" MeSH terms were also used in our analysis. We note that publications from cities called "Lebanon" in the United States have been excluded from our studies; we only included papers from Lebanon, the Middle Eastern country. Furthermore, in the case of Palestine, the West Bank and Gaza strip affiliations were used. Figure 1 represents an algorithm-based visual representation of the methodological approach used in this study.

Dynamic keyword and authorship analysis

VOS Viewer (Visualization of Similarities Viewer) program was used to assess the co-occurrence of author and MeSH keywords, with a threshold of 10 minimum keyword co-occurrences. This program was used to generate a bibliographic map containing clusters of suicide- and self-mutilation-related keywords with the average year of occurrence using the overlay visualization option. In addition, the program screened for possible collaborations between organizations with a minimum threshold of three common publications. This approach will help us assessing partnerships and research collaborations between Arab institutions and countries. These bibliographic maps present a dynamic explorative analysis of suicide-related topics and focus in the Arab world between 2004 and 2019.

Statistical analysis

In order to assess the correlation between the number of publications and the other socio-economic variables previously mentioned, linear regression was carried out on SPSS (Statistical Package of Social Sciences) version 23.

## Results

The average population size, GDP, and suicide and self-harm-related DALY of the 22 Arab countries between 2004 and 2019 are presented in Table 1. A total of 209,452 articles were published in Arab countries in this time frame, of which 198 (0.09%) were related to suicide and self-mutilation (Table 1).

**Table 1:** Average population, GDP (in $ billion), and percentage of suicide and self-mutilation related DALY for Arab countries (2004-2019). * West Bank and Gaza. GDP- Gross domestic product.

Country	Av. Population	Av. GDP (Billion $)	Av. DALY (%)
Algeria	37,359,438	160.65	1.00
Bahrain	1,249,091	27.05	1.80
Comoros	718,660	0.94	0.61
Djibouti	866,421	1.59	0.56
Egypt	86,266,141	215.90	0.83
Iraq	32,084,985	151.16	0.82
Jordan	7,885,521	28.09	0.74
Kuwait	3,234,030	125.03	0.98
Lebanon	5,626,327	38.89	1.14
Libya	6,245,227	52.76	1.51
Mauritania	3,683,546	4.23	0.38
Morocco	33,134,513	91.13	1.25
Oman	3,538,838	59.49	0.83
Palestine*	4,205,124	9.64	0.69
Qatar	1,962,132	122.42	1.78
Saudi Arabia	28,762,301	569.45	0.66
Somalia	12,636,382	4.22 **	0.33
Sudan	36,084,250	63.26	0.51
Syria	19,046,416	31.92 ***	0.49
Tunisia	10,812,039	41.23	0.64
United Arab Emirates	7,980,982	312.86	1.48
Yemen	24,224,626	28.89	0.62

In addition, 32,625 suicide and self-mutilation articles were published worldwide; Arab countries contributed only 0.61% of the total suicide-related publications (Table 2). To put these results into context, other regions of the world that encompassed low to middle-income countries, similar to the Arab world, were assessed for suicide-related publications. South America, which includes Argentina, Bolivia, Brazil, Chile, Colombia, Ecuador, Guyana, Paraguay, Peru, Suriname, Uruguay, and Venezuela, contributed to 1.76% (576 articles) of the total suicide-related publications. The Far East Asian countries (Brunei, Cambodia, China, Hong Kong, Macau, Japan, North Korea, South Korea, Mongolia, Siberia, Taiwan, East Timor, Malaysia, Laos, Indonesia, Myanmar, Singapore, Philippines, Thailand, and Vietnam) contributed to 7.94% (2,593 articles) suicide-related articles. Therefore, South America and Far East Asia had significantly higher contributions to suicide than the Arab world, albeit having similar economic characteristics. A similar approach for comparison was used by Daou et al. [17].

**Table 2:** Suicide-related subjects/fields of Arab countries with ten or more publications.

Article Subjects	Egypt	Iraq	Jordan	Kuwait	Lebanon	Morocco	Saudi Arabia	Tunisia	UAE
Self-Mutilation	1	0	0	0	2	3	3	1	1
Suicidal Ideation	11	4	8	3	15	6	10	4	4
Suicidal Assisted	1	0	1	3	1	0	2	0	0
Suicidal Attempted	9	2	6	5	9	11	14	7	5
Suicidal Completed	1	0	0	0	0	0	0	0	0

In Arab countries, the percentage of suicide and self-mutilation-related articles ranged from 0.02% in Oman to 0.25% in Lebanon and Iraq. However, numerous countries, including Syria, Djibouti, and Mauritania, had no published studies on suicide and self-mutilation between 2004 and 2019. Egypt had the highest number of suicide and self-mutilation-related publications with 33 publications, followed by Saudi Arabia with 31 publications (Table 3).

**Table 3:** Number of suicide and self-mutilation publications, total publications, and percentage of suicide and self-mutilation publications, out of the total publications for the Arab countries (2004-2019). * West Bank and Gaza.

Country	Publications	Total Publications	Percentage
Algeria	2	4,345	0.05
Bahrain	2	1,582	0.13
Comoros	-	42	-
Djibouti	-	101	-
Egypt	33	57,976	0.06
Iraq	12	4,885	0.25
Jordan	16	11,841	0.14
Kuwait	11	6,071	0.18
Lebanon	29	11,709	0.25
Libya	-	1,047	-
Mauritania	-	120	-
Morocco	19	8,085	0.24
Oman	1	5,201	0.02
Palestine*	1	1,126	0.09
Qatar	5	9,168	0.05
Saudi Arabia	31	56,357	0.06
Somalia	-	111	-
Sudan	8	3,295	0.24
Syria	-	1,438	-
Tunisia	16	15,505	0.10
United Arab Emirates	12	8,176	0.15
Yemen	-	1,271	-
Total	198	209,452	0.09

**Figure 2:**
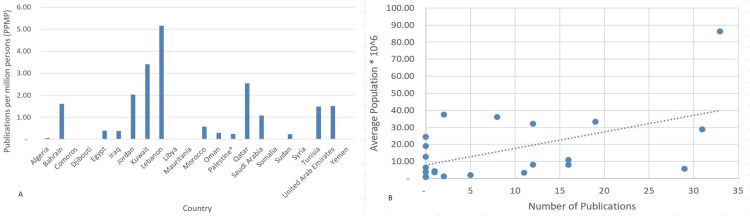
Suicide and self-mutilation publications per million persons (PPMP) values for the Arab countries (2004-2019). A: Bar graph B: Scattered plot * West Bank and Gaza.

After normalizing to population size to avoid bias, the publications per million persons (PPMP) were calculated using the average population sizes for each country during this period. Countries with smaller populations, such as Lebanon and Kuwait, proved to be more productive than larger Arab publishers in Egypt and Saudi Arabia. Lebanon ranked first in terms of PPMP (5.15 PPMP), followed by Kuwait with 3.40 publications per million persons (Figure 2A, 2B).

**Figure 3:**
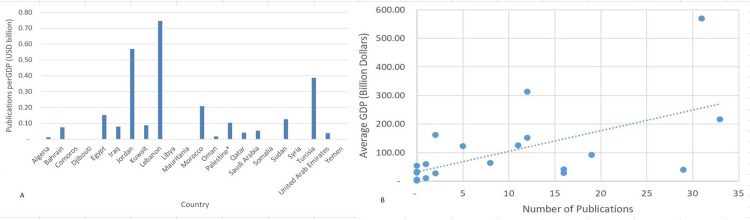
Suicide and self-mutilation publications per Gross Domestic Product (in USD billions) for the Arab countries (2004-2019). A: Bar graph B: Scattered plot * West Bank and Gaza.

Given that research usually requires funding, the economic status of each country should also be taken into consideration. The GDP (USD billion) is a measure essentially used to assess and compare national economies. For that, the average number of published articles per GDP was calculated using the average GDP of Arab countries. Lebanon proved to have the highest number of suicide and self-mutilation-related publications per billion USD with 0.75 publications per GDP, while Jordan ranked second with 0.57 publications per GDP (Figure 3A, 3B).

**Figure 4:**
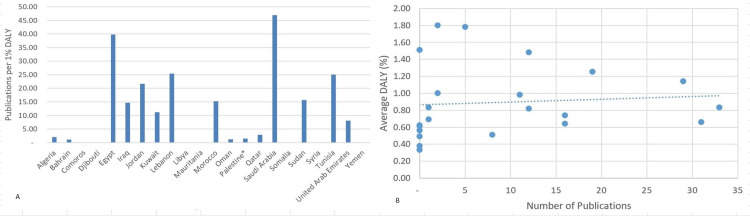
Suicide and self-mutilation publications per 1% self-harm-related DALY (2004-2019). A: Bar graph B: Scattered plot * West Bank and Gaza.

DALYs related to self-harm differed amongst the 22 Arab countries, ranging from 0.33% in Somalia to 1.80% in Bahrain. However, Bahrain had the lowest number of publications per 1% DALY with 1.11, while Saudi Arabia ranked first with 46.97 publications per 1% DALY (Figure 4A, 4B). Countries with a higher DALY are expected to have thoroughly engaged researchers in suicide and self-mutilation fields. Linear regression indicates relatively strong positive correlations between the number of suicide and self-mutilation publications with both the GDP (r = 0.597; p=0.003) and the population size (r = 0.528; p=0.011). Additionally, no significant correlation was found between the number of suicide and self-mutilation-related publications and the DALYs (r = 0.085; p=0.707).

**Figure 5:**
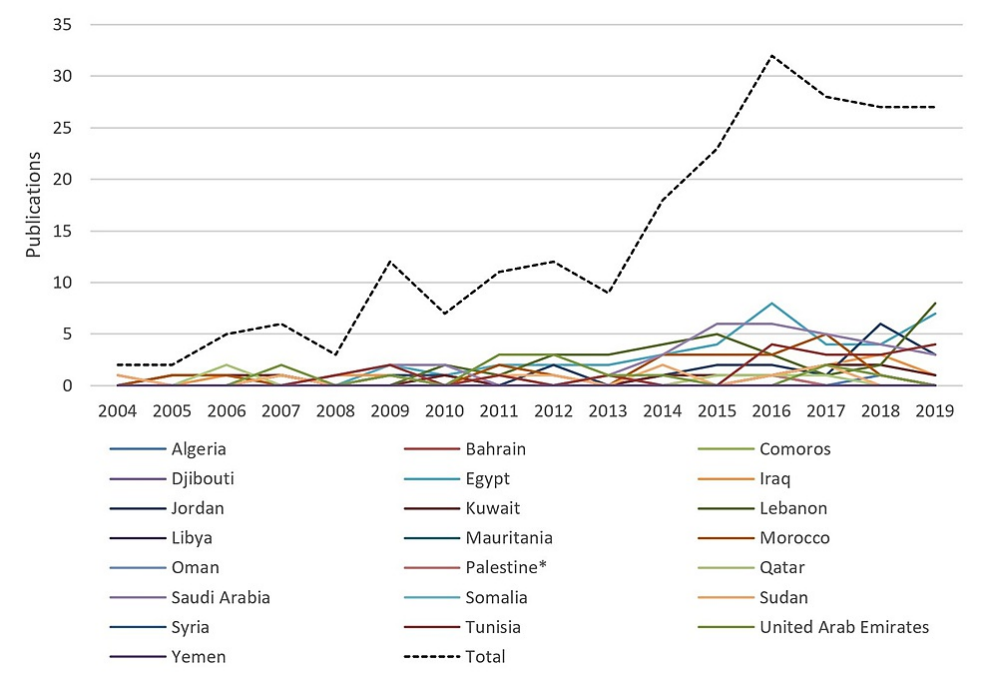
Publications related to suicide and self-mutilation in Arab countries (2004-2019). * West Bank and Gaza.

Publications on suicide showed an improvement in numbers between 2004 and 2019, with the highest increase between 2013 and 2016 (Figure 5). Total research productivity in the Arab world increased by 160% in the last ten years [12]. However, research productivity concerning suicide and self-mutilation was lower than the Arab world's total average, with an average increase of 137.48% between 2004 and 2019. 

**Figure 6:**
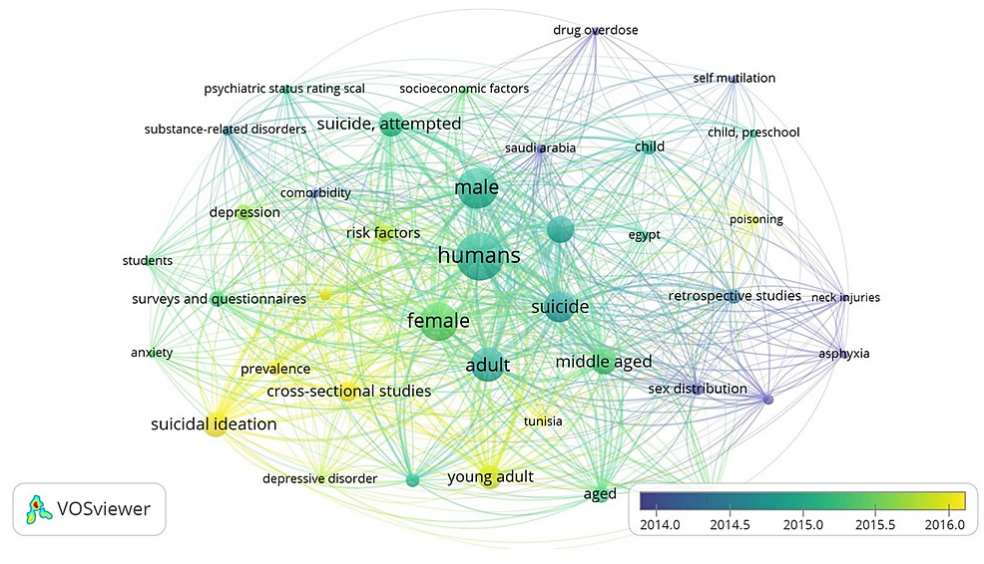
Co-occurrence of suicide- and self-mutilation-related author and MeSH keywords between 2004 and 2019.

According to figure 6, keywords are aggregated into three main clusters. Cluster 1contains 19 items: adult, anxiety, comorbidity, cross-sectional studies, depression, depressive disorder, female, humans, Lebanon, male, mental disorders, prevalence, psychiatric status rating scale, risk factors, students, substance-related disorders, suicidal ideation, suicide attempted, survey and questions. Cluster 2 contains 17 items: Adolescent, age distribution, age 80 and over, asphyxia, child, preschool, drug overdose, Egypt, middle-aged, neck injuries, poisoning, retrospective studies, Saudi Arabia, self-mutilation, sex distribution, suicide, Tunisia. Cluster 3 is only composed of 1 item: socio-economic factors. Overlay visualization showed that early research focused on neck injuries, asphyxia, drug overdose, and retrospective studies (average year: 2014) and recent research focused on the depressive disorder, suicidal ideation, anxiety, students, poisoning, and cross-sectional studies (average year: 2016).

Assessment of co-authorship between organizations in suicide- and self-mutilation-related publications in the Arab world revealed modest partnerships and collaborations. Collaborations with more than three common publications between Arab universities and organizations were absent. The only country with three or more co-authored publications was Lebanon: 12 co-authored documents were published with the partnership of Lebanese University, Lebanese International University, and University Saint-Esprit Kaslik.

## Discussion

Research articles in the Arab world are not equally distributed among countries. For instance, Egypt, Saudi Arabia, and Lebanon published 33, 31, and 29 articles, respectively, accounting for 44.49% of the total articles published by all Arab countries (Table 2). In other words, the other 19 countries combined approximately published half of suicide and self-mutilation articles.

Normalizing the number of publications by different indicators appeared to be of extreme importance, as suggested by several studies [17-21]. These indicators are the population size, average GDP, and DALYs. As expected, Lebanon, a country with an average population of only 5.6 million and a relatively low GDP, has topped the list when normalizing to population size (Figure 2) and GDP (Figure 3), and Saudi Arabia ranked first when normalizing to DALY (Figure 4). We found that GDP and population size exhibited a strong positive correlation with the number of publications. Surprisingly, countries like Bahrain and Qatar, where suicide burdens were the highest, only published 2 and 5 articles, respectively, within the adopted timeframe.

Research activity in the Arab world

In general, there has been an increase in research activity on suicide and self-mutilation in the Arab world in the last 16 years: three and five articles were published in 2004 and 2005 respectively, compared to 34 in 2017, and then maintaining a relatively steady but slightly decreasing number with 32 and 30 respectively in 2018 and 2019 (Figure 5). These numbers are still low compared to other areas and countries worldwide since only 0.61% of the total suicide and self-mutilation publications worldwide originated in Arab countries.

Lebanon has the third-highest scientific impact with 29 publications. Most of these articles were published from 2017 to 2019 (Figure 5), leading us to assume that concerns over suicide and self-harm have been rising.

Causes of the low research activity

It is certain that "brain drain," lack of global recognition of Arab universities, the Arab spring, the lack of funding, and the socio-economic crises are some of the main reasons for low research activity in general [22]. However, suicide, in particular, might have some additional causes, including its consideration as a shameful act by family and religion. Arab cultures emphasize the role of family bonds, such as the importance of honouring family. However, these values could also lead to communication problems within the family and lower suicide tolerance [23]. The sensitivity of this subject can be seen in our results as there is only one article approaching completed suicide (Table 2).

Interestingly, Carpiniello et al., 2017 suggest a reciprocal relationship between suicide and stigma, emphasizing that stigmatization on its own is a risk factor for suicide [24]. Moreover, a major cause of the poor research outcome is the lack of intraregional and interregional collaborations. As previously mentioned in the results, only Lebanon had three or more institutionally co-authored publications. Co-authorship in Lebanon was confined to 3 Universities only. Surprisingly, no single multinational collaboration was to be found in a region compromised of 22 countries. Thus, a pan-Arabic strategy targeting suicide-related research is climacteric.

Analysis of the articles' content

Curiously, MeSH keywords aggregated into three main clusters. Cluster 1 included 19 items, from which we cite: adult, depression, students, Lebanon, and substance-related disorder. For instance, Kazour et al. (2015) discovered that heroin-dependent subjects in Lebanon were at higher depression and suicide risk when a family history of suicide and multiple drug uses were present [25]. Cluster 2 included 17 items, from which we mention: adolescent, age distribution, sex distribution, neck injuries, poisoning, and suicide. A good proportion of articles discussed suicide at a young age: 16% of examined adolescents had suicidal thoughts, 22% of university students in countries with a Muslim majority had suicidal ideation with a difference regarding sex, and 8.6% had already attempted suicide [26]. Cluster 3 is only composed of1 item: socio-economic factors.

Interestingly, the trends of topics have emphatically improved, highlighting previously untackled causes of suicide. On the one hand, early research addressed drug overdose, neck injury, asphyxia, and retrospective studies (Fig. 5); these articles focused on the influence of culture and legislation on the methods used in suicide [27] and on the epidemiological aspects of suicide. These topics showed that suicide trends vary by region, country, and especially gender. On the other hand, recent suicide research focused on depressive disorders, suicidal ideation, anxiety, students, poisoning, and cross-sectional studies (Fig. 5). A particular subject of interest was the association between suicide and mental health. Studies found that 77.5% of suicide attempters had mental health disorders, and 27.5% of suicide attempters had schizophrenia in particular, and 36.6% were depressed [28]. To sum it up, we can safely admit that research in the Arab is improving, quantitatively and especially qualitatively; over the years, articles targeting suicide are becoming more forthright, asking the right questions and addressing the issue directly.

Articles' subject repartition

Interestingly, only 11 articles related to self-mutilation were published in the nine most productive Arab countries regarding suicide and self-mutilation publications (Table 2). NSSI is not given the importance it deserves in clinical research for being a risk factor for suicide [9]. Additionally, 65 articles discussed suicidal ideation, and 68 discussed attempted suicide. Focusing on these two aspects is of crucial importance since 18.49% of the general population experienced suicidal ideation at one point in their life, and 7.6% of students had a history of suicide attempts [29], knowing that prior suicide attempt is the most significant risk factor for suicide according to the WHO [1].

Taboos in the Arab world

In general, research is underprivileged in the Arab world: 189 papers per 1 million people were produced in the Arab world; other countries averaged four times more. Only 4 of the 22 Arab countries (Qatar, Tunisia, Lebanon, and Kuwait) exceeded the world average. Nevertheless, a promising increase in publications can be noted, mainly due to the rise in concern for research and student research (SR) implementation into the Arab world [30]. Student research showed remarkable benefits in other parts of the world, such as a more informed career choice and increased research productivity. However, taboo subjects such as mental health are rarely discussed and are frequently a source of shame in cultures. Consequently, the media and the scientific community must collaborate to shift the social discernment of suicide and mental health issues from a sinful act to a treatable disease.

Limitations

Our study is not without limitations. First, PubMed was the only database used to procure the released publications. Secondly, no criteria were used to classify articles according to their importance, type, and number of citations. Finally, we did not include ongoing research works and research published in the year 2020. All these factors might lead to an underestimation of the actual number of publications in the Arab world.

## Conclusions

Despite their prominent occurrence in the Arab World, suicide and self-mutilation are underprivileged in research work. A modest but promising research trend towards previously under-reported causes of suicide has been noticed. However, inter and intraregional collaborations between Arab institutions are limited. Experts and scientific groups should make efforts to shift society's perception of mental health issues and suicide. Therefore, Arab countries should invest more time and resources in clinical and social research, which would eventually decrease the burden of suicide and enhance medical research output at both the country and institutional levels.
